# DAPK Promoter Methylation and Bladder Cancer Risk: A Systematic Review and Meta-Analysis

**DOI:** 10.1371/journal.pone.0167228

**Published:** 2016-12-01

**Authors:** Lihe Dai, Chong Ma, Zhensheng Zhang, Shuxiong Zeng, Anwei Liu, Shijie Tang, Qian Ren, Yinghao Sun, Chuanliang Xu

**Affiliations:** Department of Urology, Changhai Hospital, Second Military Medical University, Shanghai, China; West China Second Hospital, Sichuan University, CHINA

## Abstract

**Background:**

Methylation of tumor suppressor gene promoter leads to transcription inactivation and is involved in tumorigenesis. Several studies demonstrate a potential association between the Death-Associated Protein Kinase (DAPK) gene promoter methylation and bladder cancer risk, tumor stage and histological grade. Due to inconsistent results of these studies, we performed this meta-analysis to ascertain the association.

**Methods:**

Studies were retrieved from the PubMed, Embase, Web of Science and the Cochrane Library databases. Study selection and data extraction were executed by two reviewers independently. Meta-analysis was performed using Stata 13.0 and Review Manager 5.3 software.

**Results:**

A total of 21 articles involving 15 case control and 8 case series studies were included in this meta-analysis. DAPK promoter methylation was associated with bladder cancer risk (OR: 5.81; 95%CI = 3.83–8.82, P<0.00001). The frequency of DAPK promoter methylation was equal in bladder cancer tissue and paired adjacent normal tissue (OR: 0.87; 95%CI = 0.31–2.48, P = 0.794). Furthermore, DAPK promoter methylation was associated with higher histological grade (OR: 1.52; 95%CI = 1.10–2.09, P = 0.011) but not associated with tumor stage (OR: 1.12; 95%CI = 0.67–1.87, P = 0.668).

**Conclusions:**

The result suggests that DAPK promoter methylation is significantly increased in bladder cancer patients compared to normal controls. DAPK promoter methylation could serve as a biomarker for bladder cancer detection and management.

## Introduction

Bladder cancer (BCa) is the 11th most commonly diagnosed cancer in the world [1]. There will be an estimated 76,960 new cases and 16,390 deaths in the US in 2016[2]. BCa is the fourth most common cancer of men and twelfth of women in the US [2]. About 75% patients are diagnosed as non-muscle invasive BCa with a high recurrence but low progression rate. The five-year survival is aproximately 90% [3, 4]. Muscle invasive BCa frequently progresses to metastasis with five-year survival less than 50% [5]. Cystoscopy with biopsy, the current standard for diagnosis and surveillance of BCa, is an invasive method that causes discomfort to patients. Urine cytology is non-invasive but yields low sensitivity in detecting low-grade lesions [6]. Recently, efforts have been made to develop biomarker for non-invasive diagnosis of BCa.

DNA methylation is involved in transcriptional silencing of tumor suppressor gene, being a common epigenetic event in early phase of tumorigenesis [7, 8]. Inactivation of tumor suppressor gene by promoter methylation has been frequently observed in BCa [9–11]. The Death-Associated Protein Kinase (DAPK) gene is located on chromosome 9p34. It encodes Ca+/calmodulin-regulated serine/threonine kinase, which induces apoptosis and suppresses tumor growth [12, 13]. DAPK promoter methylation was reported to be associated with varies cancers including BCa [14]. Down regulation of DAPK expression by promoter methylation was observed in both tissue and cell lines of BCa [15, 16]. DAPK promoter methylation was detected in the urine and blood of BCa patients, making it a potential non-invasive diagnostic biomarker [17–19]. In addition, DAPK promoter methylation was associated with tumor stage and histological grade [16, 20–22]. However, there were inconsistent results among different studies in evaluating the association between DAPK promoter methylation and BCa risk, tumor stage and histological grade. Thus, we conducted a meta-analysis to ascertain the association.

## Materials and Methods

This meta-analysis was designed according to the latest version of PRISMA checklist for meta-analysis (**S1 File**).

### Publication selection

Studies were identified via a search of PubMed, EMBASE, Web of Science and the Cochrane Library databases updated on 8, March, 2016 using the following key words: (‘‘methylation” or ‘‘hypermethylation”) and (‘‘bladder cancer” or ‘‘bladder neoplasm” or ‘‘bladder tumor” or ‘‘bladder carcinoma” or ‘‘bladder carcinogenesis” or “urothelial carcinoma”) and (‘‘DAPK or ‘‘Death-associated protein kinase”). References of these publications were manually reviewed in order to retrieve additional studies.

### Inclusion and exclusion criteria

Studies were reviewed by two authors (LHD and CM) independently. Studies met the following inclusion criteria were eligible for the meta-analysis: (1) Purpose of study was to evaluate DAPK promoter methylation in either tissue, urine or blood of BCa patients and normal controls or to assess the association between DAPK promoter methylation and tumor stage or histological grade, (2) types of sample from BCa patients and normal controls should be homogeneous, (3) Detection method of DAPK promoter methylation was based on methylation specific polymerase chain reaction (MSP), (4) Articles published in English. When several studies with overlapping data were observed, only those with a larger sample size were included. Studies without sufficient data after contacting with the original author were excluded.

### Data collection

Information extracted from each included study were author, year of publication, ethnicity of individuals involved, type of sample, method for methylation examination, pathological stage, histological grade, frequency of DAPK promoter methylation in BCa patients, normal controls and adjacent normal bladder tissue. The normal controls were defined as those without a historical or current diagnose of BCa, including healthy people, those with benign urological diseases and those with tumors of the other kinds. Studies assessing DAPK promoter methylation in both tissue and urine of BCa patients and normal controls were treated as two. Histological grade ≥2 was defined as high grade, otherwise low grade. Tumor stage ≥2 T2 was classified as high stage, otherwise low stage. Data collection was carried out by two reviewers independently (LHD and CM). Different opinions were settled by discussion.

### Quality assessment of individual study

Quality of individual study was assessed in accordance with the Newcastle-Ottawa Scale (NOS) assessment[23] separately by two authors. Articles contain case control studies were scored according to selection, comparability and exposure. Assessment was made by scoring with stars ranging from zero to nine. Article scored with six or more stars were qualified.

### Statistical Analysis

The pooled odds ratios (ORs) and corresponding 95% confidence intervals (CIs) of different studies were calculated to compare dichotomous statistics between studies. To assess heterogeneity across the studies, Cochrane’s Q test[24] and *I*^*2*^ statistic[25, 26] were calculated. If the studies were shown to be homogeneous with P>0.05 and *I*^*2*^ values < 50%, the fixed-effects model (the Mantel-Haenszel method) were selected. Otherwise, a random-effects model (the DerSimonian and Laird method) was applied. In addition, a sensitivity analysis was performed to assess the stability of the results. The potential publication bias was examined in a funnel plot visually and the degree of asymmetry was tested by Egger’s test[27]. This meta-analysis was performed using the software STATA version 13.0 (Stata Corporation, TX, USA) and Review Manager 5.3(Cochrane Collaboration, Oxford, UK). All P-values were based on two-sided tests and a P<0.05 was considered statistically significant.

## Results

### Study selection and characteristics

**Fig 1:**
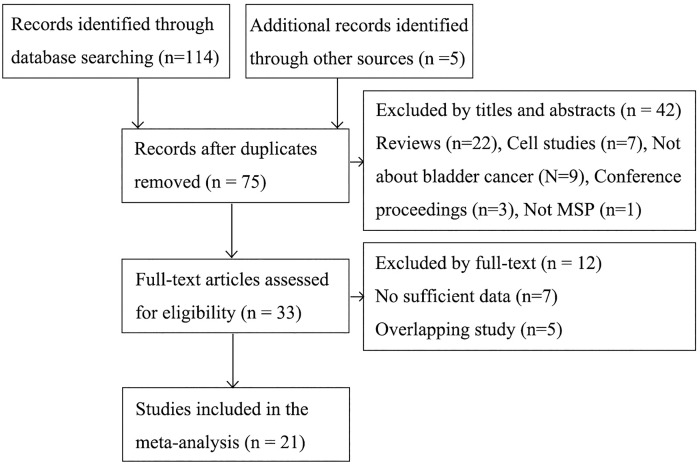
Selection of studies in the meta-analysis.

The strategies for study selection and the results were presented in **Fig 1**. The study by Motlagh et al [28] examined DAPK promoter methylation in the tissue from BCa patients and the blood from healthy individuals, thus excluded. All of the 7 articles without sufficient data after contacting the authors were case series studies. The result of NOS assessment showed all case control studies were qualified for this meta-analysis (**S1 Table**). Finally, 21 articles [15–22, 29–41] were enrolled including 15 case control and 8 case series studies.

The frequency of the DAPK promoter methylation was evaluated in a total of 1,247 samples from BCa patients and 405 from normal controls in 15 case control studies. Among them nine involved Caucasians and six Asians. Six studies applied MSP method and nine studies used qMSP. MethyLight was classified as qMSP method [42]. DAPK promoter methylation was assessed in tissue only, in urine only, in both tissue and urine and in blood in 14, 3, 2 and 2 studies respectively. DAPK promoter methylation was assessed in 233 paired BCa tissue and adjacent normal bladder tissue in five studies. In addition, the association between DAPK promoter methylation and tumor stage and histological grade were evaluated in 13 case control and 4 case series studies. Characteristics of included studies were listed in **Table 1**.

**Table 1:** Characteristics of the Included Studies

First author	Year	Ethnicity	Sample type	Control type	Method	Sample size	Case		Control		Low stage		High stage		Low grade		High grade	
							M+	U	M+	U	M+	U	M+	U	M+	U	M+	U
Chan-t^a^[21]	2002	Asian	tissue	non-BCa	MSP	102	60	42	0	7	-	-	-	-	-	-	-	-
Chan-u^b^[21]	2002	Asian	urine	non-BCa	MSP	22	10	12	0	17	8	10	2	2	5	4	5	8
Friedrich[15]	2004	Caucasian	urine	non-BCa	MethyLight	37	8	29	0	20	4	11	4	18	-	-	-	-
			tissue	ANT	qMSP	33	9	24	19	14	-	-	-	-	-	-	-	-
Nakagawa[29]	2005	Asian	tissue	non-BCa	MSP	63	18	45	1	7	10	34	5	14	-	-	-	-
Christoph[30]	2006	Caucasian	tissue	non-BCa	qMSP	110	81	29	4	16	57	9	24	20	-	-	-	-
Yates[31]	2006	Caucasian	urine	non-BCa	qMSP	35	2	33	3	66	-	-	-	-	-	-	-	-
Yates[32]	2007	Caucasian	tissue	ANT	qMSP	23	4	19	1	22	14	56	7	14	1	19	20	51
Ellinger[33]	2008	Caucasian	blood	non-BCa	qMSP	42	1	41	0	39	0	11	1	30	-	-	-	-
Jarmalaite[34]	2008	Caucasian	tissue	non-BCa	MSP	58	16	42	1	1	10	28	6	14	1	9	15	33
Wolff[20]	2008	Caucasian	tissue	non-BCa	qMSP	253	11	242	0	8	5	184	6	58	1	98	10	144
			tissue	ANT	qMSP	43	0	43	3	40	-	-	-	-	-	-	-	-
Hellwinkel[35]	2008	Caucasian	tissue	ANT	qMSP	39	24	15	26	13	-	-	-	-	-	-	-	-
Brait[36]	2008	Caucasian	tissue	non-BCa	qMSP	32	7	25	0	5	85	70	29	26	-	-	-	-
Sobti[37]	2010	Asian	tissue	non-BCa	MSP	103	39	64	4	44	24	33	15	31	-	-	-	-
Jablonowski[17]	2011	Caucasian	blood	non-BCa	MSP	42	27	15	0	36	-	-	-	-	21	9	6	6
Chen-t^a^[19]	2011	Asian	tissue	non-BCa	qMSP	210	114	96	0	2	-	-	-	-	30	28	84	68
Chen-u^b^[19]	2011	Asian	urine	non-BCa	qMSP	30	8	22	2	17	6	19	2	2	3	9	5	13
Vinci[18]	2011	Caucasian	urine	non-BCa	qMSP	108	27	81	10	95	-	-	-	-	15	50	12	31
			tissue	ANT	qMSP	85	41	44	26	59	-	-	-	-	22	28	19	16
Maruyama[38]	2001	Caucasian	tissue	-	MSP	98	4	94	-	-	2	40	2	54	-	-	-	-
Tada[16]	2002	Asian	tissue	-	MSP	55	16	39	-	-	-	-	-	-	0	5	16	34
Friedrich[39]	2005	Caucasian	tissue	-	MethyLight	105	25	80	-	-	-	-	-	-	1	22	24	58
Neuhausen[40]	2006	Caucasian	tissue	-	MSP	88	28	60	-	-	5	8	23	52	1	2	27	58
Park[22]	2010	Asian	tissue	-	MSP	64	18	46	-	-	9	40	9	6	6	17	12	29
Jarmalaite[41]	2010	Caucasian	tissue	-	MSP	21	4	17	-	-	-	-	-	-	2	3	2	14

Abbreviation: non-BCa, non-bladder cancer; ANT: adjacent normal tissue; M+, methylated; U, unmethylated.

^a^tissue of BCa patients and control in studies evaluating DAPK promoter methylation in both tissue and urine.

^b^urine of BCa patients and control in studies evaluating DAPK promoter methylation in both tissue and urine.

### The correlation of DAPK promoter methylation and BCa

#### 1. DAPK promoter methylation in BCa patients and normal controls with stratified analysis

**Fig 2:**
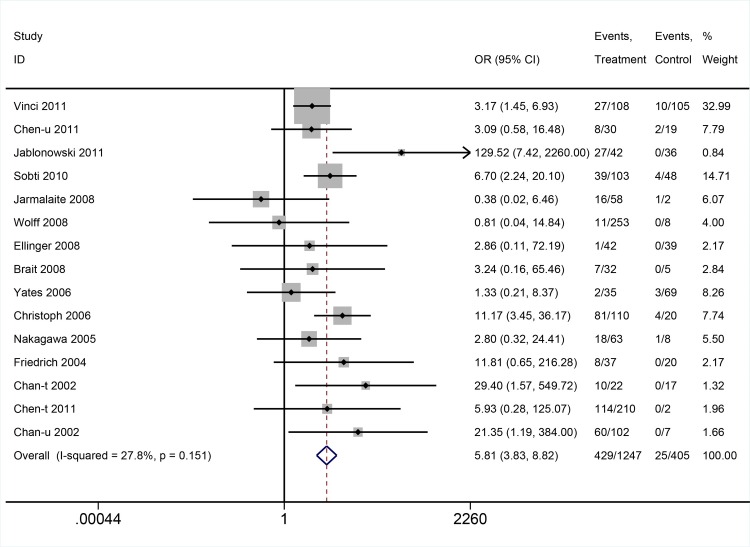
The pooled OR from 15 studies including 1,247 samples form BCa patients and 405 from normal controls. OR: 5.81; 95%CI = 3.83–8.82, P<0.00001.

Altogether, DAPK hypermethylation was associated with increased risk of BCa (OR: 5.81; 95%CI = 3.83–8.82, P<0.00001, fixed-effects model) (**Fig 2**). We validated the result in stratified analysis by type of sample, ethnicity and detection method (**Table 2**).

**Table 2:** Stratified analysis of the association between DAPK promoter methylation and bladder cancer risk

Variables	p^a^	OR	95%CI	Heterogeneity		
				X	P	I
**Total**	15	5.81	3.83–8.82	19.38	0.151	27.8%
**Ethnicity**						
**Asian**	6	6.80	3.25–14.24	3.07	0.689	0.0%
**Caucasian**	9	4.02	1.56–10.33	15.67	**0.047**^**b**^	49.0%
**Method**						
**qMSP**	9	4.03	2.40–6.78	6.57	0.584	0.0%
**MSP**	6	9.95	4.76–20.81	10.80	0.055	53.7%
**Material**						
**Urine**	5	3.89	2.13–7.09	4.03	0.402	0.8%
**Blood**	2	38.23	5.55–263.37	3.18	0.075	68.5%
**Tissue**	8	5.90	3.09–11.26	7.95	0.337	11.9%

^a^Number of comparisons.

^b^Random-effects model.

#### 2. DAPK promoter methylation in paired BCa tissue and adjacent normal bladder tissue

**Fig 3:**
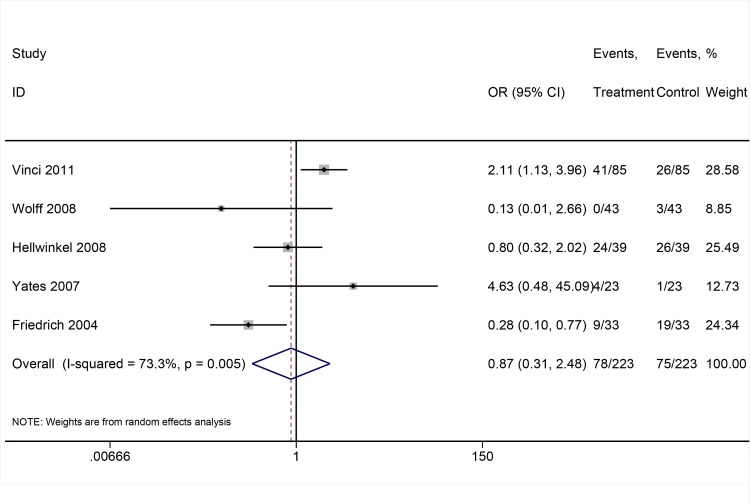
The pooled OR from 5 studies including 223 paired BCa tumor tissue and adjacent normal bladder tissue. OR: 0.87; 95%CI = 0.31–2.48, P = 0.794.

Then, we investigated the level of DAPK promoter methylation in BCa tissue and adjacent normal bladder tissue. In pooled meta-analysis, the frequency of DAPK promoter methylation was equal in paired BCa tissue and adjacent normal bladder tissue (OR: 0.87; 95%CI = 0.31–2.48, P = 0.794, random-effects model) (**Fig 3**).

#### 3. The association between DAPK promoter methylation and tumor stage or histological grade of BCa

**Fig 4:**
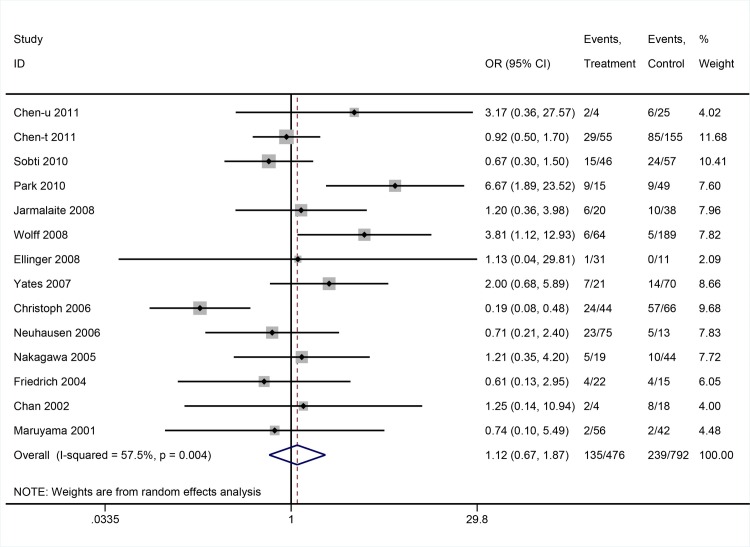
The pooled OR from 14 studies assessing the association between DAPK promoter methylation and tumor stage. OR: 1.12; 95%CI = 0.67–1.87, P = 0.668.

**Fig 5:**
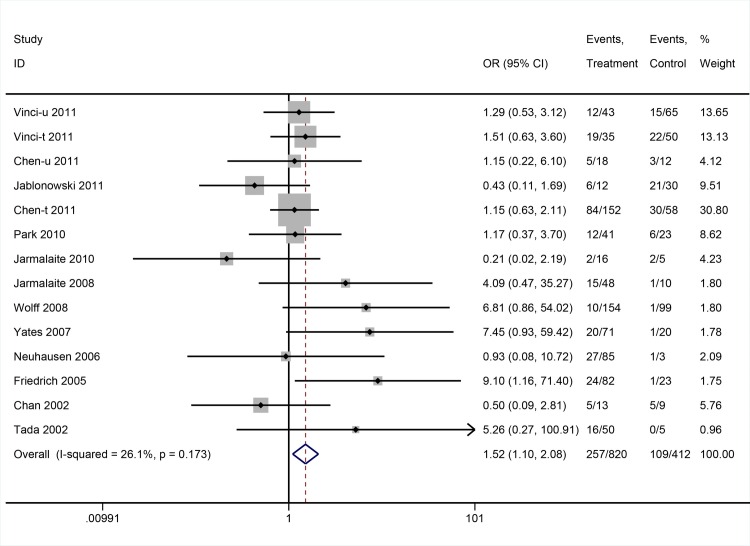
The pooled OR from 14 studies assessing the association between DAPK promoter methylation and histological grade. OR: 1.52; 95%CI = 1.10–2.09, P = 0.011.

There was no association between DAPK promoter methylation and tumor stage (OR: 1.12; 95%CI = 0.67–1.87, P = 0.668, random-effects model) (**Fig 4**). On the contrary, DAPK promoter methylation was associated with higher histological grade (OR: 1.52; 95%CI = 1.10–2.09, P = 0.011, fixed-effects model) (**Fig 5**).

#### 4. Sensitivity Analyses

**Fig 6:**
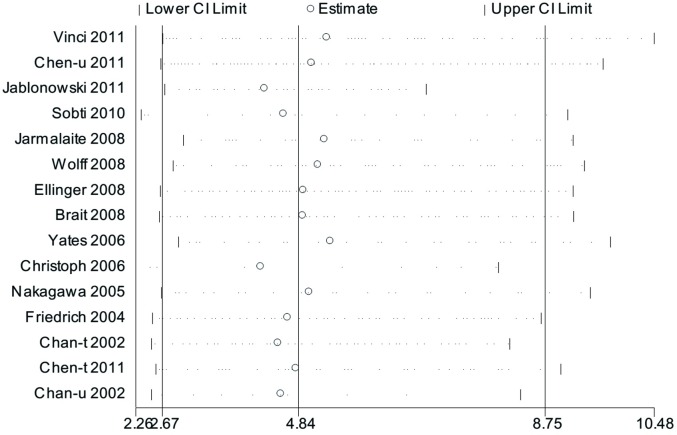
Sensitivity analysis from 15 studies comparing DAPK promoter methylation in BCa patients and normal controls.

**Fig 7:**
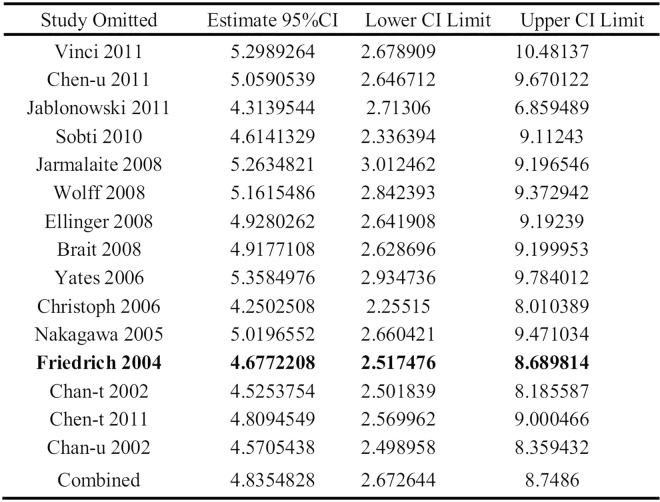
Sensitivity analysis from 14 studies assessing the association between DAPK promoter methylation and histological grade.

Sensitivity analysis showed that the pooled ORs ranged from 4.25(95%CI: 2.26–8.01) to 5.36 (95%CI: 2.93–9.78) (**Fig 6**), indicating that none of the studies dramatically changed the pooled ORs. The results of the other meta-analysis turned out to be reliable and stable after sensitivity analysis (**S1 Fig**) except for studies of the association between DAPK promoter methylation and histological grade (**Fig 7**). In omitting Friedrich’s [39] study, which was the only one applying MethyLight technology in this set, the result turned out that DAPK promoter methylation was not associated with histological grade (OR: 1.38; 95%CI: 0.99–1.91, P = 0.054, fixed-effects model). The same result was seen when random-effects model was applied (OR: 1.38; 95%CI: 0.89–2.14, p = 0.155).

#### 5. Publication Bias

**Fig 8:**
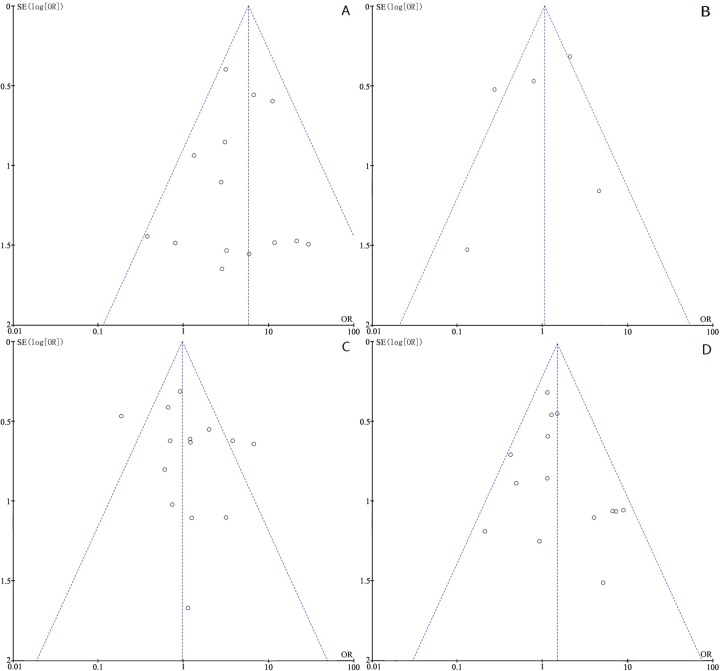
The funnel plots were virtually symmetrical, suggesting there were no publication bias in this meta-analysis. (A)The funnel plot from 15 studies comparing DAPK promoter methylation in BCa patients and normal controls. (B)The funnel plot from 5 studies with paired BCa tissue and adjacent normal bladder tissue. (C)The funnel plot from 14 studies assessing the association between DAPK promoter methylation and tumor stage. (D)The funnel plot from 14 studies assessing the association between DAPK promoter methylation and histological grade.

Funnel plots of all the studies above were listed in **Fig 8**. No publication bias was observed, as the shape of the funnel plots seems to show no evident asymmetry in each meta-analysis. It is further validated by the Egger’s test (P>0. 05) (data not shown).

## Discussion

DNA methylation plays an important role in epigenetic transcriptional control and genome stability [43]. Methylation induced silencing of tumor suppressor gene has been documented in varies types of cancer [14, 44]. In our meta-analysis, DAPK promoter methylation, detected either in urine, blood or tissue, in Asian or Caucasian, using MSP or qMSP method, was significantly associated with increased risk for BCa.

Previous studies demonstrated that the frequency of DAPK promoter methylation was associated with tumor stage [22, 37] and histological grade [17, 20], while others reported no association [15, 30, 35, 40]. This meta-analysis indicated that there was no significant association between DAPK promoter methylation and stage. Though it appeared that DAPK promoter methylation was associated with higher histological grade under fixed-effects model, the result changed in sensitivity analysis and after adopting random-effects model. Thus, we may carefully draw the conclusion whether there was an association between DAPK promoter methylation and histological grade of BCa. In addition, DAPK promoter methylation frequency was equivalent in paired BCa tissue and adjacent normal bladder tissue. We may postulate that DAPK promoter methylation is an early or pre-cancerous event in BCa tumorigenesis. Based on these findings, DAPK promoter methylation may serve as an effective biomarker for early diagnose and surveillance of BCa. Due to the reversibility of DNA methylation [45], therapeutic target based on DAPK promoter methylation may be developed.

The DAPK gene is involved in the p53-dependent apoptosis pathway and has been found to be inactivated via promoter methylation in lung cancer [46, 47]. DAPK promoter methylation was associated with decreased expression of DAPK protein and mRNA in BCa tissue [16, 37]. It was also observed in BCa cell lines to be associated with mRNA down-expression [48]. DAPK mRNA and protein re-expressed after treated with 5-aza-2'-deoxycytidine, inhibiting growth and inducing apoptosis of BCa cell line [48, 49]. Arsenic exposure induced DAPK promoter methylation and transcription inactivation in urothelial cancer [50, 51]. Additionally, smoking increased the risk of DAPK promoter methylation in BCa patients [18, 37]. In this sense, we may postulate that there is a correlation between DAPK promoter methylation and carcinogen exposure of BCa, which further supports its role in BCa tumorigenesis.

Our study has several limitations. First, most of the studies were retrospective, information and selection biases and the other confounders may not be fully controlled. Second, seven studies were excluded due to insufficient data in evaluating the association between DAPK promoter methylation and tumor stage and histological grade, which may lead to publication bias and influence the reliability of our conclusion. Third, as regard to source of control, we failed to distinguish between the healthy and those with different types of benign urological diseases. Fourth, while only studies published in English were included, studies of high quality in the other languages may be excluded.

## Conclusions

In conclusion, our meta-analysis demonstrated that DAPK promoter methylation is significantly associated with BCa tumorigenesis. Further well-designed prospective studies with larger sample size may provide more valid evidence of the role of DAPK promoter methylation in BCa tumorigensis and disease management.

## Supporting Information

S1 File PRISMA 2009 Checklist. (DOC)

S1 Table Quality assessment of case control studies according to the Newcastle-Ottawa Scale. (DOCX)

S1 Fig Sensitivity analysis of the other three meta-analysis in this paper. S1A Fig. Sensitivity analysis from studies of DAPK hypermethylation in matched bladder cancer tissue and adjacent normal tissue. S1B Fig. Sensitivity analysis from studies of association between DAPK hypermethylation and tumor stage. S1C Fig. Sensitivity analysis from studies of association between DAPK hypermethylation and tumor grade.
(DOCX)
