# Supplementary material for: DAPK Promoter Methylation and Bladder Cancer Risk: A Systematic Review and Meta-Analysis
Source: PLoS One. 2016 Dec 1;11(12):e0167228. doi: 10.1371/journal.pone.0167228 (PMC5132202; doi:10.1371/journal.pone.0167228)
Supplement: S1 Table — (DOCX) [file pone.0167228.s002.docx]

**DAPK Promoter Methylation and Bladder Cancer Risk: A Systematic Review and Meta-analysis**

Lihe Dai ^¶^, Chong Ma ^¶^, Zhensheng Zhang, Shuxiong Zeng, Anwei Liu, Shijie Tang, Qian Ren, Yinghao Sun, Chuanliang Xu*

|  |  | 1 | 2 | 3 | 4 | 5 | 6 | 7 | 8 | Score |
| --- | --- | --- | --- | --- | --- | --- | --- | --- | --- | --- |
| Chan et al. | 2002 | ★ | ★ | ★ | ★ | ★★ | ★ | ★ | ★ | 9 |
| Friedrich et al. | 2004 | ★ |  | ★ | ★ | ★ | ★ | ★ | ★ | 7 |
| Nakagawa et al. | 2005 | ★ |  |  | ★ | ★ | ★ | ★ | ★ | 6 |
| Christoph et al. | 2006 | ★ | ★ |  | ★ | ★ | ★ | ★ | ★ | 7 |
| Yates et al. | 2006 | ★ | ★ | ★ | ★ | ★★ | ★ | ★ | ★ | 9 |
| Ellinger et al. | 2008 | ★ |  |  | ★ | ★ | ★ | ★ | ★ | 6 |
| Jarmalaite et al. | 2008 | ★ | ★ |  | ★ | ★ | ★ | ★ | ★ | 7 |
| Wolff et al. | 2008 | ★ | ★ |  | ★ | ★★ | ★ | ★ | ★ | 8 |
| Brait et al. | 2008 | ★ | ★ |  | ★ | ★ | ★ | ★ | ★ | 7 |
| Sobti et al. | 2010 | ★ | ★ |  | ★ | ★ | ★ | ★ | ★ | 7 |
| Jablonowski et al. | 2011 | ★ |  | ★ | ★ | ★ | ★ | ★ | ★ | 7 |
| Chen et al. | 2011 | ★ | ★ |  | ★ | ★ | ★ | ★ | ★ | 7 |
| Vinci et al. | 2011 | ★ | ★ | ★ | ★ | ★★ | ★ | ★ | ★ | 9 |

**Table S1. Results of NOS assessment for case control studies.**

1. Adequate definition of cases; 2. Representativeness of cases; 3. Selection of controls; 4. Definition of controls; 5. Control for important factor; 6. Ascertainment of Exposure; 7. Same method to ascertain for cases and controls; 8. Non-response rate.
