# Supplementary material for: DAPK Promoter Methylation and Bladder Cancer Risk: A Systematic Review and Meta-Analysis
Source: PLoS One. 2016 Dec 1;11(12):e0167228. doi: 10.1371/journal.pone.0167228 (PMC5132202; doi:10.1371/journal.pone.0167228)
Supplement: S1 Fig — S1A Fig. Sensitivity analysis from studies of DAPK hypermethylation in matched bladder cancer tissue and adjacent normal tissue. S1B Fig. Sensitivity analysis from studies of association between DAPK hypermethylation and tumor stage. S1C Fig. Sensitivity analysis from studies of association between DAPK hypermethylation and tumor grade. (DOCX) [file pone.0167228.s003.docx]

**S1A Fig.** Sensitivity analysis from studies of DAPK hypermethylation in matched bladder cancer tissue and adjacent normal tissue

**S1B Fig.** Sensitivity analysis from studies of association between DAPK hypermethylation and tumor stage

**S1C Fig.** Sensitivity analysis from studies of association between DAPK hypermethylation and tumor grade
